# Food Advertisement and Dietary Choices in Adolescents: An Overview of Recent Studies

**DOI:** 10.3390/children10030442

**Published:** 2023-02-24

**Authors:** Anastasia Tsochantaridou, Theodoros N. Sergentanis, Maria G. Grammatikopoulou, Kyriakoula Merakou, Tonia Vassilakou, Eleni Kornarou

**Affiliations:** 1 Department of Public Health Policy, School of Public Health, University of West Attica, 196 Alexandras Avenue, 11521 Athens, Greece; 2 Department of Rheumatology and Clinical Immunology, Faculty of Medicine, School of Health Sciences, University of Thessaly, Biopolis, 41110 Larissa, Greece

**Keywords:** influencers, obesity, Instagram, online gaming, food marketing

## Abstract

Adolescents are exposed to food marketing through many routes, including television, movies, videos, print media, online games, and social media. The interplay between exposure to unhealthy food advertisements and food choices by adolescents is a field of special interest given the ongoing evolution of social media trends and marketing strategies. The purpose of this review was to synthesize the scientific findings in the last five years (2017–2022) regarding the possible influence of nutrition-related advertisements through television, social media, or video games on the choice and consumption of unhealthy foods and drinks in adolescents. Nineteen studies were included in this review. Adolescents exposed to unhealthy food and beverage advertising showed high desire and intention to consume the advertised foods, as evidenced by the majority of included studies. The effects of advertisements are reinforced by peer pressure and influencers and interact with socioeconomic, biological, and environmental factors. Food marketing represents part of the obesogenic environment of the present time.

## 1. Introduction

Food marketing is an important factor influencing eating behavior, especially in children and adolescents [1]. Food marketing refers to “any communication designed to increase recognition, appeal and/or consumption of specific food products, brands and services” [2].

Children and adolescents are an important part of the market, as effective marketing to them can create early positive relationships and long-term consumer and brand relationships that extend into adulthood [3]. Indeed, children and adolescents can buy food directly, which makes it a “primary market”; furthermore, they can determine family purchasing decisions, thus constituting an “influence market”. Finally, their loyalty to the brand at a young age promises continued sales into adulthood, i.e., as later consumers they represent a “future market” [4].

Unhealthy food patterns have been noted among adolescents, whereas dietary intake often does not meet the standards of international recommendations [5,6]. Therefore, the World Health Organization (WHO) has called for restrictions on the marketing of certain products, such as tobacco products and unhealthy foods/drinks to children and young adolescents [7]. Junk food advertisements have been described as a detrimental factor shaping junk food consumption in adolescents, along with interactions with environmental factors [8].

Adolescents are exposed to food marketing through many routes, including television, movies, videos, advergames, product placements, cross-promotions, product packaging, athlete and celebrity endorsements, charitable donations by food and beverage companies, billboards, radio, print media, internet, mobile phones, and social media; advertising can take many forms, including images, videos, and games that promote specific brands [9,10,11,12].

Regarding the persuasive techniques used, those linked to advertising focus on the taste, smell, texture, nutritional quality, competitiveness, uniqueness, and novelty of the product, while emotional appeals are also occasionally used, such as in the focus on family ties in some TV commercials [13]. Other techniques that are used by TV commercials to make food products appealing to kids include faster pacing, the use of cartoon characters, and the choice of scenarios inspired by everyday life [14].

Adolescents use a variety of new media platforms, including social networks, messaging, interactive games, mobile phones, and virtual 3D environments, a fact with a crucial role in identity development, peer relationships, and learning [15,16]. At the same time, the growth of peer-to-peer social networking platforms is creating what some scholars consider a powerful new form of “mass interpersonal persuasion.” By shaping and delivering a “convincing experience” at high speed to many users, behaviors and attitudes can be changed on a massive scale [17]. In this environment, digital food marketing brings novel opportunities and poses new challenges. It has been estimated that children and adolescents are exposed to food marketing on social media to an impressive extent, namely 30 and 189 times per week for children and adolescents, respectively [18].

The three most common food-related categories advertised to children and adolescents, according to Busse et al., are sweets, sugary drinks, and fast-food restaurants. Similarly, Amson et al., examining digital food and beverage-related content, noted that the most frequently promoted food and beverage categories were fast food, energy drinks, and sweets [19]. In a study by Bathrellou et al., nutritional value analysis revealed that the majority of advertised foods on TV during scheduled children’s television shows fell into the unhealthy food category, being high in sugar and fat (e.g., confectionary snacks and chocolate products) [14].

The interplay between exposure to unhealthy food and beverage advertisements and food choices by adolescents is a rapidly evolving field given the ongoing evolution of social media trends and marketing strategies. In light of the above, the purpose of this review was to synthesize the most recent scientific findings in the past five years regarding the possible influence of nutrition-related messages through television, social media, or video game advertisements on the choice and consumption of unhealthy foods and drinks in adolescents, which then lead to overweight and obesity risk.

## 2. Materials and Methods

Eligible articles were limited to studies conducted in humans (quantitative or qualitative) examining exposure to unhealthy food/drink advertisements on television, social media, or video games as a factor affecting food consumption, purchase, eating and nutritional choices (main meals or snacking), overeating, overweight, or obesity in adolescents.

Exclusion criteria were the following: studies merely reporting exposure rates to food and drink advertisements in adolescents; studies with a focus on the effect of advertisements on the dietary choices of parents or the family in general; studies involving advertising messages viewed in theaters, print media, or movies at the cinema; studies related to programs with the aim of reducing adolescent exposure to unhealthy food and beverage advertisements; studies centered on the use of school-based programs in order to implement a policy to improve and prevent obesity; research on populations with image perception disorder, depressive symptoms, or suicidal ideation; and systematic reviews or meta-analyses.

Studies referring exclusively to children up to 12 years of age or exclusively to young adults and students were excluded, while studies referring to adolescents in either middle (the period between 14 and 16 years of age) or late adolescence (17–19 years of age) were included. Studies on adolescents with the joint inclusion of children were allowed to be included.

No sample size restriction was applied. Items published in English within the last five years were considered.

The search was performed in the PubMed/MEDLINE, Scopus, and ScienceDirect databases with combinations of keywords (“obesity”, “adolescent”, “adolescence”, “teenager”, “food advertising”, “food advertisement”, “food choice message”) for the 1 January 2017 to 1 July 2022 time period.

## 3. Results

### 3.1. Selection and Overall Description of Studies

This search led to 359 items. Of these, 202 items were rejected after reading the title. After studying the abstract and methodology, 141 items were then rejected, and 16 were selected for further evaluation of the full text of the publication. There were 3 items that emerged after studying the bibliographic references of the retrieved articles, which were subsequently included.

Therefore, a total of 19 studies were included in this review (Table 1).

A total of 19 published articles, were included in the review. The studies took place in Europe (Spain [34], Belgium [25,36], Ireland [30], the UK [26,27], The Netherlands [31], Turkey [33]), the USA [20,21,23,24,28,29,32,37], Uruguay [38], and Australia [22,35].

Cervi et al. [20] and Gesualdo and Yanovitzky [24] examined the effect of advertising messages on the consumption of sugary drinks. Critchlow et al. [27] focused on the consumption of unhealthy foods, while the remaining articles examined the effect of nutritional advertising messages on the consumption of unhealthy beverages and foods. In the majority of articles, the data collection process was carried out either electronically online or in public and/or private schools. Secondly, data collection was carried out in specialized spaces, such as laboratories or via email or the use of computers [21,22,29,30]. Finally, another context used to collect information was the home environment [23].

The age of participants, in almost all studies, ranged from 11 to 18 years, except for four of them that, in addition to adolescents, included children in the sample [21,22,23,31]. The upper age limit in three studies was 19 years of age [26,27,36].

In longitudinal cohorts, the age of onset was 8–12 years; the duration in both of them was 2 years [21,31].

In this literature review, in addition to the cross-sectional and longitudinal studies mentioned, three randomized clinical trials [23,32,34], three qualitative studies [24,25,38], and two experimental studies were included. The sample sizes ranged from 21 to 8708 adolescents. In all studies, the sample included subjects from both sexes.

### 3.2. Measurement of Variables in Individual Studies

**Demographic and socio-economic variables.** In all studies, demographic data and information on the socio-economic status and education level of the mother or parents were collected using questionnaires, usually in electronic form. Two studies, specifically those by Thomas et al. [26] and Critchlow et al. [27], used the Index of Multiple Deprivation (a measure of the relative deprivation of an area) in their methodology, while Gascoyne et al. (2021) used the Socio-Economic Index for Areas and the Index of Relative Socio-Economic Disadvantage to calculate the socio-economic status of the area [35].

**Anthropometric variables.** To calculate and assess the degree of obesity/overweight of the subjects to be examined, the Body Mass Index (BMI) was mainly used. Regarding anthropometric characteristics, height and weight in four studies [21,29,31,33] were measured by specialized personnel according to protocols. In five studies [20,24,27,34,36], those measurements were reported by the adolescents themselves or by their guardians. Powell et al. [21] implemented the dual-energy X-ray absorptiometry method as an additional measurement in order to measure the body fat mass and lean mass indices.

**The advertisement–nutrition interplay.** Data were collected on behaviors related to nutrition, the consumption of mainly fast food or foods characterized as unhealthy, and the consumption of sugary drinks such as soft drinks.

In addition, information was collected on Internet and social media use, including adolescent engagement with social media sites (such as Facebook, Instagram, and YouTube) and interactions with different commercial food and beverage companies.

The influence of advertised food and beverages on teenagers’ purchase and consumption choices was evaluated in different ways in different surveys. Thus, in the article by Powell et al. [21], advertising data were based on individual television program ratings; scores were measured in target rating point units for specific subgroups of the population. Monthly target rating points were aggregated to the brand level and then categorized across food categories using a product classification code [21].

In two studies [20,24], the variable of interest was the adolescent’s sensitivity to the influence of advertising. Gesualdo and Yanovitzky [24] examined three potential mediators of the association between advertising sensitivity and sugar-sweetened beverage preference and consumption: self-efficacy in limiting sugary beverage consumption, perceived norms regarding SSB consumption by peers, and the attitude towards sugar-sweetened beverages. In addition, a measure of the adolescent’s degree of exposure to an obesity-promoting environment was created.

Additionally, in the study by Qutteina et al. [25], participants were trained to take screenshots of any food-related images they received and/or associated with social media, while in 11 studies [22,25,26,27,28,30,31,33,34,35,37] questionnaires were used to collect information related to the effect of advertising messages on the consumption of unhealthy drinks and foods. Ares et al. used interviews with experienced researchers for this purpose [38].

It is worth noting that Gearhardt et al. used the functional magnetic resonance neuroimaging method in order to evaluate teenagers’ preference for advertisements [29]; Murphy et al. used a Tobii-T60 eye-tracking monitor [30].

In terms of the type of media used to promote food and beverages, social media was most common in the studies included in this literature review. However, Powell et al. [21], Thomas et al., [26], Fleming-Milici and Harris [28], and Fernández-Escobar et al. [34] investigated television viewing in addition to social media sites, while Dikmen et al. [33] examined only the effect of advertising messages from television.

### 3.3. Main Findings of the Studies

Almost all studies found a positive association between food advertisement and adolescent obesity or overweight and/or the choice to consume unhealthy foods and beverages, with the exception of three studies [20,21,31] that did not confirm the relationship. Specifically, Powell et al. [21] showed that exposure to advertisements for soft drinks and sugary drinks was significantly associated with a higher frequency of their consumption among young people, but noted that the association between fast-food advertising and its consumption was not confirmed. Exposure to cereal advertising was significantly associated with young adolescents’ BMI, but exposure to fast-food and soft drink advertising was not. Results on obesity revealed that children’s exposure to cereal advertising was associated with body fat percentage. The same was true for fast-food advertising. In contrast, exposure to sugar-sweetened beverage advertising was marginally significantly associated with body fat percentage. Similarly, this lack of relationship between advertising messages and the consumption of unhealthy foods was shown by Smit et al. [31].

Gesualdo and Yanovitzky focused on the role of peer influence. Specifically, the researchers reported that advertising-vulnerable adolescents were more likely to report a preference for sugar-sweetened beverages if the advertising led them to perceive that their peers consumed these sugar-sweetened beverages regularly [24].

### 3.4. Interactions with Gender, Age, Socio-Economic Status, and “Dose”

Regarding between-gender differences, three studies dealt with that parameter [25,27,33]. Qutteina et al. reported that girls were more likely to share images of food from Instagram and Snapchat. On the other hand, boys were more likely to share images of food on YouTube and Facebook [25]. However, Dikmen et al. pointed out that girls were more influenced by television advertisements and tended to buy the advertised foods more than boys [33]. Similarly, Critchlow et al. reported that gender was an important factor, with girls more likely than boys to react positively to all parameters studied for the advertisements, except perceived health. The main finding of this study was that adolescents reacted positively to advertisements for foods high in fat, salt, and sugar, with the majority having positive reactions to each advertisement. This included positive perceptions of branding (e.g., perceived popularity and appeal) and advertisement design (e.g., fun and age group appeal), as well as behavioral impact (e.g., temptation to try) [27].

Critchlow et al. referred to age-specific effects. Specifically, positive reactions had key correlations with age; younger teenagers were more likely to report that advertisements were aimed at their age group and were more tempted to try the promoted brands [27].

Living in deprived neighborhoods seems to influence the effect of food and beverage advertisements on the consumption of these products, which may also be due to teenagers’ high exposure to them [26]. In addition, Cervi et al. addressed race, with black study participants being more influenced by advertisements [20].

Two studies [28,37] focused on the potential dose–response relationship between exposure duration and choice of foods to consume. Specifically, the findings of Fleming-Milici and Harris showed greater risk of engagement during high versus moderate television viewing across all food categories, suggesting a possible dose–response relationship [28]. Moreover, regarding screen format, the study indicated that exposure to television advertising may have a greater influence on engagement than time spent on digital media overall [28]; the study by Harris et al. also supported the same notion regarding television viewing [37].

## 4. Discussion

This review highlights that adolescents exposed to unhealthy food and beverage advertising showed high desire and intention to consume the advertised foods, as evidenced by the majority of included studies [20,21,22,23,24,25,26,27,28,29,30,32,33,34,35,36,37,38].

Powell et al. reported that exposure to advertisements for soft drinks and sugary drinks was significantly associated with higher consumption among young people, whereas exposure to cereal and fast-food advertising correlated with body fat percentage [21]. The association between advertisements and the consumption of unhealthy foods has been consistently reported in research published before the study period (2017–2022) [39,40,41,42], as well as in the recent systematic review by Kucharczuk et al. [43]. Notably, the systematic review and meta-analysis of 18 studies showed that acute exposure to food advertising for unhealthy food and nonalcoholic beverages increased food intake in children but not in adults [44]. The advertising of unhealthy foods and beverages to adolescents has been recognized as a major contributor and factor of great importance in the genesis, expansion, and persistence of obesity [39,42,44].

Digital marketing includes promotional activities undertaken through websites, social networking sites, e-mail, texts on mobile phones, applications, and online games. Online marketing advertisements are often qualitatively different from traditional adverts. Rather than passively receiving messages, online advertisements actively engage children through advertising platforms (e.g., games with branded content) and/or through invitations to be brand ambassadors (e.g., encouraging children to contact friends for a product) [45,46]. Therefore, digital marketing could be more influential than traditional marketing due to the characteristic of peer approval and the lack of explicit advertising cues presented in some forms of digital media [47]. Digital marketing can therefore engage adolescents in emotional, entertaining experiences [48]; it has been argued that adolescents are particularly vulnerable to this type of content as they are still in a phase of cognitive development [1,2]. In the world of digital marketing, “engagement” refers to the brand’s ability to interact with the consumer, to attract the consumer, and to become part of their life [49]. Guimarães et al. noted that six out of ten adverts targeting children and adolescents used abusive techniques, and almost all adverts did not present sufficient and clear information about the product; moreover, exposure to unhealthy foods was frequent [50].

A modifying role of gender emerged in this review; Dikmen et al. showed that girls were more influenced by television advertisements and tended to buy the advertised foods more than boys [33]. According to Qutteina et al., girls were more likely to share food images from Instagram and Snapchat; on the other hand, boys were more likely to share images of food on YouTube and Facebook [25]. The scoping review by Castronuovo et al. revealed that gender can play an important role in the development of different food marketing techniques and how youth respond to them; many variables could mediate the association between advertising, gender, and eating behaviors, such as weight status, nutritional knowledge, advertising techniques, media type, brand awareness, family pressures, etc. [51].

Peer interactions and peer pressure are other aspects that interact with food advertisements targeted at adolescents. Concern has been expressed that the process of “liking” posts makes social media advertising uniquely interactive and may lead teens to perceive brand names more as friends than as companies [52]. Social influences related to snacking and food choice are often driven by adolescents’ desire to “fit in” [53]. Moreover, eating with friends outside the home is part of a social ritual, which brings a sense of belonging to a group and furthermore demonstrates their competence and autonomy from the family, since they are able to actively make decisions about food [54]. According to Montaña Blasco and Jiménez-Morales, adolescents focus mainly on perceived images and the role of a drink or food in social functioning (i.e., conformity with peers) [55], a fact that might also facilitate the powerful influence of peers, advertising, and promotion on food choices. Accordingly, advertising-vulnerable adolescents were more likely to report a preference for sugar-sweetened beverages if advertising led them to perceive that their peers consume these beverages regularly [24].

On social media, advertisements posted by companies may resemble posts from friends due to context (i.e., they appear as expected with posts shared by friends) and content (i.e., company posts mimic the aesthetics of everyday consumer posts), and it remains unclear whether these marketing strategies cloud teenagers’ ability to recognize company posts as advertisements. Sherman et al. showed increased activity in the brain’s reward system among adolescents who viewed their own posts with a high number of likes versus few likes [56]. Given that the reward system is more sensitive to reward in adolescents than in adults and children [57], these findings further support the unique sensitivity of adolescents to social media advertising. Importantly, Bragg et al. reported that food adverts on Instagram were more attractive to teenagers than traditional food adverts; the mere presence of the Instagram “like” logo and comments made teens rate the adverts more favorably compared to teens who saw the same adverts without these Instagram features [32]. Worrisomely, Murphy et al. reported that teenagers responded significantly more positively to unhealthy food advertising compared to healthy food advertising. In terms of recall, recall for unhealthy food brands was almost five times higher than for healthy food brands and almost twice as high as for non-food brands [30].

Moreover, targeted marketing on social media results in different products being advertised to different populations [58]; personal preferences, online activity, and location are captured, and these data are then used to personalize and target marketing content to individual users, thereby increasing persuasive power [59]. A high frequency of targeted and curated posts on Instagram that manipulate consumers’ emotions rather than presenting objective information about their products was noted by Vassallo et al. [60].

Qutteina et al. underlined the role of influencers in social media food marketing. They found that social media focused on the consumption of non-core foods in large portions, a fact promoted by peers, marketers, and influencers alike; branded non-core/unhealthy foods were promoted through marketing strategies by influencers [25]. Similarly, in the study by Ares et al., participating adolescents recalled seeing influencers promoting food and beverages on YouTube, TikTok, and Instagram. The participants reported seeing advertisements for a wide range of products and services; the most frequently cited advertisements were for fast-food restaurants, energy drinks, soft drinks, and fortified water [38].

Online in-game advertising has become a highly sophisticated, finely tuned strategy that combines product placement, data collection, and online marketing to cultivate deep, ongoing relationships between corporate brands and individual players. Through dynamic product placement, adverts can be integrated into the game’s story and programmed to respond to a player’s actions in real time [61]. A historical example in 2005 pertains to the collaboration between Sony and Pizza Hut, where the ability to order pizza was built into the game Everquest II [62].

Despite the importance of social media in food marketing, television is an important player in the field. Examining television advertisements, Dikmen et al. reported that a total of 69.6% of adolescents stated that they were influenced by television food advertisements, and 66.4% of them purchased these foods. The preferred products were cakes, cookies, chocolate, sweet and salty snacks, and soft drinks [33]. Anderson et al. also reported that children and adolescents consumed more energy while watching a television program with food advertisements compared to television programs containing non-food advertisements [63]. Kelly et al., pooling television advertising data from 22 countries (including Australia, Canada, Chile, Costa Rica, Guatemala, Malta, Mexico, New Zealand, and Slovenia), found that children up to 18 years of age were exposed to between 1.7 (Malta) and 13.4 (Canada) food and drink adverts per hour during peak TV viewing, with a global average of 3.1 food adverts per hour. The most frequently advertised foods and beverages worldwide were carbonated soft drinks, flavored waters, chocolate, and confectionery; in general, unhealthy food items were promoted four times more than healthy foods [64]. Nevertheless, adolescents have moved towards social media, leaving television behind; the total number of food advertisements broadcast on all television stations increased by 4%, while the average exposure of adolescents to food advertising decreased by 31% due to the change in media being used by adolescents and their shift to online content sharing platforms (e.g., YouTube), video-on-demand subscription services (e.g., Netflix), and online games (e.g., Fortnite) [65,66].

Critically comparing television and online food advertisements, Demers-Potvin et al. [67] pointed out that most time spent by teenagers in Australia, Canada, Chile, Mexico, the UK and the USA was spent on digital media, with social media use varying by platform. Instagram was the most popular social media platform (52–68% by country), followed by Facebook (42–79%) and Snapchat (28–52%). Across all countries, exposure to advertising in the past 30 days was most common on television, followed by digital media and gaming platforms. A greater proportion of adolescents reported exposure to advertisements for unhealthy food or beverages on television compared to websites, social media applications, or gaming sites; however, reported exposure to TV advertising may have been higher, as it is more easily identifiable compared to digital marketing, which often uses more covert marketing techniques and is often disguised as entertainment [68,69]. In digital media, teenagers may simply be less able to distinguish adverts from other content, making marketing in these channels particularly worrisome. Additionally, Smit et al. found that children’s frequency of watching video weblogs influenced their consumption of unhealthy beverages two years later; the analyses did not, however, demonstrate a significant relationship with the consumption of unhealthy snacks [31].

Little is known about how the human brain responds to the influx of food advertisements. Individual differences in response to food advertisements may contribute to problematic food consumption. Thus, understanding of how food advertisements affect reward and attention areas of the brain is limited, as well as knowledge of how these may vary by body mass. Regardless of body weight, participants remembered food adverts better than non-food adverts [70]. In addition, lean adolescents versus obese adolescents showed greater neural response to food advertisements in areas associated with greater difficulty losing/maintaining weight; this suggests that even adolescents who do not currently show signs of pathology (e.g., had a normal weight) may be influenced by advertising in a way that could shape future dietary trends [70].

Psychological research by Harris et al. consistently showed that children and adolescents often lack the ability to resist the influence of food advertising [71]. Harris et al., argued that successful resistance to food marketing requires four conditions: active attention to advertising stimuli and an understanding of their persuasive intent; understanding of how one is influenced by these stimuli and how to resist effectively; cognitive maturity and fully developed self-regulatory abilities; and motivation to resist. From preschool through adolescence, numerous developmental barriers limit young people’s ability to satisfy these four conditions.

## 5. Limitations of the Study

This review article was limited to English language studies; no quantitative synthesis of results was attempted using meta-analysis methods. Various nationalities were included, without restrictions pertaining to geography; however, studies stemmed primarily from Europe, the USA, and Australia for the specific field and for the specific time period. Self-reporting by adolescents or their parents was frequent in the included studies; sometimes BMI was self-reported, a fact that might have underestimated true BMI values.

## 6. Areas for Further Studies

Social media is a rapidly changing field; social media that had been dominating the landscape have now been considered outdated by teenagers and young adults [72]. In this context, marketing of foods online is constantly changing, adapting to the endlessly evolving trends. Future studies should qualitatively and quantitatively address and trace any changing patterns in the association between unhealthy food advertisement in emerging social media and consumption/attitudes of adolescents while taking into account a multitude of interactions, thus integrating family-, peer-, school-, and food industry-related and societal factors, as well as the legal framework of implemented policies.

## 7. Conclusions

Social media and television food advertisements often focus on promoting the consumption of unhealthy foods and beverages, a fact associated with enhanced consumption among adolescents. The effects of advertisements are reinforced by peer pressure and influencers and interact with socioeconomic, biological, and environmental factors. Public health policies regarding unhealthy food advertisements on television and, especially, in the changing landscape of social media should be a priority. Food marketing represents part of the obesogenic environment of the present time.

## Figures and Tables

**Table 1:** Results of studies examining the association between nutrition-related messages through advertising, video games, and social media and nutritional choices and obesity/overweight/body fat in adolescents.

Authors	Country	Study Design	Way of Study Performance	Participants, Sample Size	Methods	Main Findings
Cervi et al., 2017 [20]	USA	Cross-sectional	Electronically	1080 teenagers aged 14 to 17; 48.5% boys and 51.5% girls	Each parent and adolescent completed two online surveys on diet-, physical activity-, and cancer prevention-related behaviors. A questionnaire related to the consumption of drinks (sugary, soft, energy, sports drinks) was completed and information related to the sensitivity of teenagers to advertisements (autonomous motivation, controlled motivation, barriers, self-efficacy, knowledge) was collected. BMI was self-reported.	High advertisement sensitivity was associated with greater consumption of SSBs. Adolescents with high vulnerability to advertising were more likely to consume at least one SSB daily. Non-Hispanic black adolescents were more likely to be exposed to advertisements and more likely to consume at least one SSB daily compared to their non-Hispanic white peers. However, no significant associations were found between being overweight or obese and consumption or advertisement sensitivity. No significant associations were found between BMI and sensitivity/vulnerability to advertising or BMI and daily consumption of SSBs.
Powell et al., 2017 [21]	USA	Longitudinal, 2-year, cross-sectional	The study was based on television ratings (data from NMR) linked to the ECLS-K cohort (1998–1999) and the U.S. NHANES (2003–2004).	414 children and adolescents with an onset age of 8 to 11 years of ae; 48.3% girls and 51.7% boys	Information on the frequency of fast-food, soft drink, sport drink, and fruit drink consumption in the previous seven days and the hours of TV watching per week was collected through relevant questions. BMI was measured by specialized personnel. In addition, the DXA method was used. As for NMR’s advertising data, these were based on individual TV program ratings. Scores were measured in TRP units; monthly TRPs were aggregated to the brand level and then categorized across food categories using an NMR product classification code that specified the product category.	Exposure to advertisements for soft drinks and sugary drinks was significantly associated with higher frequency of their consumption among young people, even after controlling for unobserved heterogeneity. The association between fast-food advertising and its consumption was not confirmed after controlling for unobserved heterogeneity. Exposure to cereal advertising was significantly associated with young adolescents’ BMI percentile rank, but exposure to fast-food and soft drink advertising was not. The obesity results revealed that children’s exposure to cereal advertising correlated with body fat percentage. The same was true of fast-food advertising. In contrast, exposure to SSB advertising was marginally significantly associated with body fat percentage.
Baldwin et al., 2018 [22]	Australia	Cross-sectional	About 7600 parents who were members of a group of a market research firm (McNair Ingenuity) were contacted by e-mail.	417 children and adolescents aged 10 to 16 years of age; 196 boys and 221 girls	Filling out a questionnaire on consumption of unhealthy foods and drinks, internet and social media use, (Facebook; YouTube) and interactions therein with different commercial food and beverage companies (”liking”, sharing, entering sponsored contests).	Certain online and social media behaviors were linked to higher consumption of unhealthy foods and drinks. Watching commercial content on YouTube and self-reported exposure to online food advertising were significantly associated with higher consumption of unhealthy foods and/or beverages after adjusting for demographic factors. Children who bought food online were also significantly more likely to have unhealthy eating choices.
Trude et al., 2018 [23]	USA	Randomized controlled trial	Home environment	509 children and adolescents aged 9 to 15 years of age; 227 boys and 282 girls	The intervention was divided into three phases, each lasting two months: (1) healthier drinks, (2) healthier snacks, and (3) healthier cooking methods. Social media (Facebook, Instagram, and Twitter) was used to integrate all levels of research. Recipes, news, and activities related to healthy drinks and snacks were featured daily. During each phase, guardians received a text message 3–5 times per week regarding healthy eating behavior. Study participants were not required to attend any training sessions; they were only invited to follow social media and subscribe to the messaging program (intervention group). The CIQ and BKFFQ were used, and young people’s food purchasing habits (pre-intervention and 6 to 12 months post-intervention) were measured.	Youths in the intervention group increased their purchases of healthier foods and beverages, which were 1.4 times more per week than those of youths in the comparison group. After the intervention, there was a 3.5% reduction in calories from sweets for the middle-adolescence participants in the intervention group compared to the control group. No effect on SSB consumption was observed.
Gesualdo and Yanovitzky, 2019 [24]	USA	Qualitative	National Cancer Institute’s Family Life, Activity, Sun, Health, and Eating study; electronically	1657 adolescents aged 12 to 17 years of age; 50% girls	The independent variable in all analyses was the adolescent’s susceptibility to advertising influence. The susceptibility score for each participant was obtained by averaging their responses to three items (wanting to try, thinking the advertised products will taste good, trusting the advertised messages). The dependent variables were self-reported SSB preference and SSB consumption during the previous week.	The relationship between advertising susceptibility and consumption of SSBs was mainly initiated by the perceived use of SSBs by their peers. In other words, advertising-vulnerable adolescents were more likely to report a preference for SSBs if advertising led them to perceive that their peers consumed these SSBs regularly.
Qutteina et al., 2019 [25]	Belgium	Qualitative and quantitative	Electronically	21 teenagers aged 12 to 18 years of age; 11 boys and 10 girls	Diary study with a one-week data collection period. Participants were trained to take screenshots of any food-related images (images of food, brand logos, restaurants, etc.) received and/or associated with social media. Participants were asked about the social media platform the image was found on and whether they were actively looking for the shared image or whether they inadvertently encountered it. Social media usage questions were also asked.	All participants reported high use of social media (at least three social media accounts, with some reporting up to six social media accounts). Girls were more likely to share images of food from Instagram and Snapchat. On the other hand, boys were more likely to share images of food on YouTube and Facebook. Despite more boys participating in the study, boys only shared 27% of the images. Social media focused on the consumption of non-core foods in large portions and was promoted equally by peers, marketers, and influencers.
Thomas et al., 2019 [26]	UK	Cross-sectional	Online	3348 adolescents aged 11 to 19 years of age; 49% girls and 51% boys	Deprivation was assessed by region with the IMD. Participants reported their consumption behaviors of HFSS products and healthy products such as fruit and vegetables, as well as the hours spent watching both commercial and non-commercial television services on both weekdays and weekends.	Adolescents from poor neighborhoods were more likely to consume a range of HFSS/high-fat foods and reported increased exposure to advertising for these foods.
Critchlow et al., 2020 [27]	UK	Cross-sectional	Electronically; online	3348 adolescents aged 11 to 19 years of age; 48% boys and 52% girls	Participants were shown two advertisements for HFSS food brands: (1) a fast-food brand and (2) a confectionery brand (for sweets) that were both affordable to teenagers. Data on sex, age, ethnicity, region of residence, IMD, and self-reported BMI were collected. Participants were asked to what extent each advert influenced their choice in terms of making the product attractive, healthy, or a choice for them.	Adolescents reacted positively to the two HFSS food advertisements. This included positive perceptions of branding (e.g., perceived popularity and appeal) and advert design (e.g., fun and age group appeal), as well as behavioral impact (e.g., temptation to try). Positive reactions had key associations with age (younger teenagers were more likely to report that both adverts would appeal to their age group and be tempted to try the promoted brands) and, to a lesser extent, BMI. Girls were more likely than boys to react positively to most parameters studied for both adverts.
Fleming-Milici and Harris, 2020 [28]	USA	Cross-sectional	Electronically	1564 adolescents aged 13 to 17 years of age; 46.9% boys and 53.1% girls	Participants reported their engagement with food and beverage brands on social media (“liking”, sharing, following brands), TV, or other screen time based on YRBSS questions (2018).	Widespread engagement with food and beverage brands on social media was shown among teenagers. Greater odds of engagement in high versus moderate television viewing was noted across all food categories, suggesting a greater influence of television and a possible dose–response relationship. High use of another screen format predicted teenagers’ engagement with fast-food and snack brands only.
Gearhardt et al., 2020 [29]	USA	Cross-sectional	Laboratory	171 male and female adolescents aged 13 to 16 years of age	fMRI neuroimaging, rating preference for advertisements (approximately 15 s in randomized order) typically seen by teenagers (unhealthy fast food, healthier fast food, phone), and eating a meal at a simulated fast-food restaurant. The primary outcome variable was food intake in the simulated fast-food restaurant.	Fast-food advertisements could result in greater food intake in adolescents through activation of neurobiological systems related to reward, memory, processing, and visual attention.
Murphy et al., 2020 [30]	Ireland	Two experimental studies	Schools; computer-based	151 adolescents aged 13 to 17 years of age. First study: 72 teenagers aged 13 to 14 years of age (45 girls, 27 boys). Second study: 79 teenagers aged 13 to 17 (49 girls and 30 boys).	Adolescents were requested to view the feeds of Facebook users of a similar age. They were presented with 36 profiles in random order. While viewing each profile feed, participants answered questions regarding the likelihood of sharing the posts they saw, questions about their use of digital media, and questions about their knowledge of brands, among others. At the end, participants were asked to list all the brands they remembered seeing while viewing their Facebook feeds. A Tobii-T60 eye-tracking monitor was also used in the second study.	Adolescents responded significantly more positively to unhealthy food advertising compared to non-food and healthy food advertising. In terms of recall, recall for unhealthy food brands was almost five times higher than for healthy food brands and almost twice as high as for non-food brands. In addition, adolescents recognized many unhealthy food brands and did so at about twice the rate of healthy food and non-food brands.
Smit et al., 2020 [31]	The Netherlands	Longitudinal, 2-year	Schools	453 adolescents with an initial age of 8 to 12 years; 52.5% girls and 48.8% boys	Data were obtained from the MyMovez project. Participants received the “Wearable Lab”. For seven consecutive calendar days, adolescents received daily questionnaires assessing video viewing frequency and unhealthy eating behaviors (i.e., consumption of SSBs and unhealthy snacks) through the device.	Frequency of watching vlogs influenced their consumption of unhealthy drinks two years later. The analyses did not demonstrate significant relationships with the consumption of unhealthy snacks.
Bragg et al., 2021 [32]	USA	Randomized controlled trial	Electronically	832 adolescents aged 13 to 17 years of age; 51.2% boys and 48.8% girls	Teenagers saw eight pairs of unhealthy food and drink adverts presented in random order. The teens then answered questions pertaining to how much they liked the image and how tasty they thought the featured product might be.	Food advertisements on Instagram were particularly attractive to teenagers compared to traditional food adverts, indicating that the visual appearance of adverts and context exert a strong influence on teenagers’ perceptions and choices.
Dikmen et al., 2021 [33]	Turkey	Cross-sectional	Schools	2699 teenagers aged 11 to 16 years of age; 1380 boys and 1319 girls	Adolescents were administered a questionnaire on television viewing habits and their tendency to purchase food and beverages under the influence of television advertisements.	Girls were more influenced by television advertisements and tended to buy the advertised foods more than boys. A total of 69.6% of teenagers said they were influenced by food advertisements, and 66.4% of them bought those foods. The preferred products were cakes, cookies, chocolate, sweet and savory snacks, and soft drinks.
Fernández-Escobar et al., 2021 [34]	Spain	Randomized controlled trial	Schools	857 adolescents aged 11 to 14 years of age	The intervention consisted of watching a video of a 5 min cartoon with two commercial breaks, each of which included two advertisements promoting unhealthy food and drinks with health promotion messages from HAVISA at the bottom of the commercial. Afterwards, students were presented healthy and unhealthy foods and were asked to freely choose a product. A questionnaire measured the effect of the intervention, attitudes, and the intention to consume the advertised products.	Participants exposed to unhealthy food advertising showed a high desire and intention to consume the advertised food, and the majority (about 7 in 10) chose unhealthy processed snacks over fruit or nuts. No differences were observed regarding the desire to perform physical activity or the perceived importance of a healthy diet and physical activity. This suggested that the health promotion messages used by HAVISA had little or no effectiveness in changing health-related behaviors or attitudes following this brief adolescent intervention.
Gascoyne et al., 2021 [35]	Australia	Cross-sectional	Schools	8708 adolescents aged 12 to 17 years of age; 52.6% girls and 47.6% boys	Students were asked to report how often in the past month they had seen an advert for a food or beverage product on social media, about related posts, and about how often they were consuming unhealthy foods or drinks.	Exposure to food and beverage advertising on social media was associated with high intake of unhealthy beverages, while engagement in such marketing was associated with high intake of unhealthy food and beverages. The strongest associations were seen among students who reported liking and sharing food or beverage content daily or almost daily.
Qutteina et al., 2022 [36]	Belgium	Cross-sectional	Schools	1002 adolescents aged 11 to 19 years of age; 58% girls and 42% boys	The effect of exposure to food messages was measured through thirty-five items that explored the extent to which the participant saw core and non-core food messages on social media. Participants reported the extent to which they saw food messages posted by friends or other influencers and celebrities, as well as messages posted by companies. Food intake was measured using the Flemish Food Frequency Questionnaire.	Adolescents who reported higher exposure to social media posts related to non-core/unhealthy foods were significantly more likely to also report higher consumption of them.
Harris et al., 2022 [37]	USA	Cross-sectional	Electronically	1566 adolescents aged 13 to 17 years of age; 46.9% boys and 53.1% girls	The survey included questions about television viewing, attitudes towards advertising, brand popularity, and consumption of unhealthy and healthy foods. The study also assessed teenagers’ engagement with brands on social media. Participants were asked to rate eight brands representing a range of different product types most advertised to 12–17-year-olds.	Adolescents showed a high level of preference for targeted advertising of company brands, as well as frequent consumption of these advertised products; exposure to television commercials for unhealthy products was associated with the consumption of unhealthy foods and beverages.
Ares et al., 2022 [38]	Uruguay	Qualitative	Two private secondary schools and a public health facility	209 adolescents, aged 12 to 18 years of age; 56% girls and 44% boys	Adolescents were divided into small groups consisting of 2–7 participants, who were adolescents of the same age (±1 year). A total of five researchers with previous experience in qualitative research conducted the interviews. An interview guide was used, which included questions on five main topics: social media use, exposure to digital marketing in general, and digital food marketing in particular, perceptions of digital food marketing, the perceived impact of digital marketing on food choice, and strategies to reduce the influence of digital marketing on adolescent dietary choices. The group interviews, lasting between 10 and 35 min, were recorded and then transcribed.	Participants reported seeing advertisements for a wide range of products and services. All teens reported seeing food adverts on social media or websites. The most frequently reported adverts pertained to fast-food restaurants from both large chains and small businesses. Participants recalled seeing influencers promoting food and beverages on YouTube, TikTok, and Instagram. Energy drinks, fast-food e-restaurants, soft drinks, and fortified water were the most frequent product categories. Most participants described the impact of digital marketing on actual purchase or consumption as little or non-existent. Some participants reported consuming fast food and soft drinks but did not recognize the influence of digital marketing on their decisions to do so. However, others acknowledged that advertisements they see on social media and websites influence their choices and described specific situations where they bought or consumed the advertised food or drink.

BKFFQ: Block Kids 2004 Food Frequency Questionnaire; BMI: body mass index; CIQ: Child Impact Questionnaire; ECLS-K: Early Childhood Longitudinal Study—Kindergarten; HAVISA: Hábitos de Vida Saludables en la Población Española; HFSS: high in fat, salt, and sugar; IMD: Index of Multiple Deprivation; NHANES: National Health and Nutrition Examination Survey; NMR: Nielsen Media Research; SSBs: sugar-sweetened beverages; TRP: target rating point; YRBSS: Youth Risk Behavior Surveillance System.

## Data Availability

Not applicable.
